# Prescription and dosing of urate-lowering therapy, rather than patient behaviours, are the key modifiable factors associated with targeting serum urate in gout

**DOI:** 10.1186/1471-2474-13-174

**Published:** 2012-09-16

**Authors:** Nicola Dalbeth, Meaghan E House, Anne Horne, Keith J Petrie, Fiona M McQueen, William J Taylor

**Affiliations:** 1 Department of Medicine, Faculty of Medical and Health Sciences, University of Auckland, 85 Park Rd, Grafton, Auckland, 1023, New Zealand; 2 Department of Psychological Medicine, University of Auckland, Auckland, 1023, New Zealand; 3 Department of Molecular Medicine, University of Auckland, Auckland, 1023, New Zealand; 4 Department of Medicine, University of Otago Wellington, PO Box 7343, Wellington, 6242, New Zealand

**Keywords:** Gout, Urate, Target, Allopurinol

## Abstract

**Background:**

Long term serum urate (SU) lowering to a target of <0.36 mmol/l (6 mg/dl) is recommended for effective gout management. However, many studies have reported low achievement of SU targets. The aim of this cross-sectional study was to examine the clinical and psychological factors associated with SU targets in patients with gout.

**Methods:**

Patients with gout for <10 years were recruited from primary and secondary care settings. SU target was defined as SU concentration <0.36 mmol/L at the time of the study visit. Both clinical and psychological factors associated with SU target were analysed. The relationship between SU target and measures of gout activity such as flare frequency, tophi, work absences, and Health Assessment Questionnaire-II was also analysed.

**Results:**

Of the 273 patients enrolled into the study, 89 (32.6%) had SU concentration <0.36 mmol/L. Urate-lowering therapy (ULT) use was strongly associated with SU target (p < 0.001). In those patients prescribed ULT (n = 181), allopurinol dose, patient confidence to keep SU under control, female sex, and ethnicity were independently associated with SU target. Other patient psychological measures and health-related behaviours, including adherence scores, were not independently associated with SU target in those taking ULT. Creatinine clearance, diuretic use, age, and body mass index were not associated with SU target. Patients at SU target reported lower gout flare frequency, compared with those not at target (p = 0.03).

**Conclusions:**

ULT prescription and dosing are key modifiable factors associated with achieving SU target. These data support interventions focusing on improved use of ULT to optimise outcomes in patients with gout.

## Background

Gout is characterised by deposition of monosodium urate (MSU) crystals in the joints and other tissues. The central strategy for management of gout is long term serum urate (SU) lowering, which leads to dissolution of MSU crystals, prevention of acute gout flares, and regression of tophi [1]. A number of studies have demonstrated that long term SU lowering below a target of 0.36 mmol/l (6 mg/dl) is required to achieve these outcomes [2-5].

Based on these studies, a ‘treat to serum urate target’ approach has been recommended in a number of gout treatment guidelines [6,7]. However, studies from many countries have shown that gout management is frequently poor, with low achievement of treatment targets [8-11]. A variety of factors may contribute to under-treatment of gout [12]. Potential patient factors include medical co-morbidities, concomitant medications, attitudes to disease and therapy, and poor adherence to urate-lowering therapy (ULT) [10,13,14]. Medical practitioner factors may include prescription and adequate dosing of ULT [8]. To date, these factors have not been systematically explored in relation to achieving optimal SU targets. Understanding these factors is important to guide treatment strategies to improve management of gout. The aim of this study was to examine the clinical and psychological factors associated with SU target <0.36 mmol/L in patients with gout.

## Methods

Patients with gout for less than 10 years were recruited by community advertising and through primary and secondary care clinics in Auckland and Wellington, New Zealand. As in other regions of the world, gout management is frequently suboptimal in New Zealand [8,15]. The inclusion criteria for this study were: a previous physician diagnosis of acute gout as defined by the Wallace classification for acute gout [16]; first attack of gout within the last 10 years; and ability to complete forms in English and provide written informed consent. Patients did not have an acute flare at the time of their study visit. The New Zealand Multi Regional Ethics Committee approved this study.

Participants attended a study visit which included a comprehensive clinical assessment. The following data were recorded: demographic data (age, gender, ethnicity), gout history (confirmation of diagnosis, disease duration, frequency of gout flares, gout treatments), medical history, examination, questionnaires (including Health Assessment Questionnaire (HAQ)-II, Brief Illness Perception Questionnaire (B-IPQ) [17], confidence in gout treatment questionnaire and a medication adherence score related to ULT (completed only by those patients taking ULT) [17]), and laboratory tests (including SU and creatinine).

Patient self-reported confidence in gout treatment was assessed using 0 (not at all confident)-10 (very confident) Likert scales, which covered aspects of gout management including confidence to follow recommended diet, take ULT regularly, have blood tests and keep blood urate level under control. These items were adapted from a diabetes-specific Multidimensional Diabetes Questionnaire [18].

Data were analysed using SPSS (v19, SPSS Inc., Chicago, IL). Means with standard deviations (SD) and percentages were used to describe the clinical characteristics of participants. SU target was defined as SU concentration <0.36 mmol/L at the time of the study visit. T-tests and Chi square analysis was used to compare the group of patients at SU target with those who were not. Forward stepwise logistic regression analysis was used to determine the independent variables associated with SU target in those prescribed ULT, with factors included in the model with p < 0.15 in the univariate analysis. The measures of gout activity were pre-specified as flare frequency, presence of tophi, work absences, and activity limitation as assessed by the HAQ-II. All tests were two tailed and P < 0.05 was considered statistically significant.

## Results

### SU target and clinical characteristics

A total of 273 patients attended the study visit. There were 89 (32.6%) patients with serum urate level <0.36 mmol/L (‘at SU target’ group). The clinical characteristics are shown for all patients in Table 1. Patients were predominantly middle-aged men treated in primary care. ULT was prescribed in 181 (66.3%) of the entire group. Allopurinol was the most frequently used ULT, in 177 patients. Four patients received probenecid monotherapy, and no patients were taking febuxostat, benzbromarone or recombinant uricase. In univariate analysis, SU target was associated with female sex, prescription of ULT, and allopurinol dose (Table 1). No relationship was observed between SU target and disease duration, body mass index, diuretic use, serum creatinine, or creatinine clearance. Anti-inflammatory treatments such as colchicine or non-steroidal anti-inflammatory drugs were not associated with SU targets, and rates of secondary care management were not different between groups.

**Table 1:** Clinical characteristics

	**At SU target (<0.36 mmol/L)**	**Above SU target (≥0.36 mmol/L)**	**p**
	**n = 89**	**n = 184**	
Age, years, mean (SD)	61 (14)	58 (16)	0.09
Male sex, n (%)	52 (58%)	143 (77.7%)	0.002
Māori or Pacific ethnicity, n (%)	15 (17%)	49 (26.6%)	0.09
Disease duration, years, mean (SD)	5.6 (4.8)	5.0 (3.0)	0.22
Treatment in secondary care, n (%)	16 (18%)	52 (28.2%)	0.07
Body mass index, kg/m^2^, mean (SD)	30.4 (6.8)	31.6 (6.5)	0.16
Diuretic use, n (%)	26 (29%)	54 (29.3%)	1.0
Any ULT, n (%)	74 (83%)	107 (58.1%)	<0.001
Allopurinol use, n (%)	74 (83%)	103 (56.0%)	<0.001
Allopurinol dose for patients on allopurinol, mg/day, mean (SD)	235 (87)	194 (98)	0.004
Probenecid use, n (%)	2 (2%)	6 (3.2%)	1.0
Colchicine use, n (%)	26 (31%)	61 (33.2%)	0.58
Non-steroidal anti-inflammatory use, n (%)	14 (16%)	43 (23.4%)	0.16
Serum urate, mmol/L, mean (SD)	0.27 (0.07)	0.47 (0.09)	<0.001
Serum creatinine, μmol/l, mean (SD)	99 (77)	110 (62)	0.23
Creatinine clearance, ml/min, mean (SD)	68 (27)	70 (30)	0.51

### Psychological measures, health-related behaviours and SU target

Although there was no difference between groups in confidence to follow dietary advice, patients at SU target reported greater confidence to take gout medications regularly and to keep SU concentrations under control (Table 2). The perceived ability of medications to control gout (BIPQ treatment control score) and understanding of disease (BIPQ understanding score) were higher in those at SU target, compared with those not at target (Table 2). Other illness perception scores and health-related behaviours did not differ between the two groups (Table 2 and data not shown). In univariate analysis of those prescribed ULT, adherence scores were higher in patients at SU target, compared to those who were not at SU target (Table 2).

**Table 2:** Psychological measures, health-related behaviours and SU target

	**At SU target (<0.36 mmol/L)**	**Above SU target (≥0.36 mmol/L)**	**p**
	**n = 89**	**n = 184**	
Confidence to keep serum urate under control, mean (SD)	7.4 (2.4)	6.7 (2.7)	0.02
Confidence to follow diet, mean (SD)	7.5 (2.3)	7.4 (2.3)	0.76
Confidence to have blood tests at recommended frequency, mean (SD)	9.4 (1.1)	9.0 (2.0)	0.06
Confidence to take gout medications regularly, mean (SD)	9.5 (1.4)	9.0 (1.7)	0.03
BIPQ treatment control score, mean (SD)	8.2 (2.4)	7.4 (2.8)	0.02
BIPQ personal control score, mean (SD)	6.1 (3.1)	5.5 (3.1)	0.18
BIPQ understanding score, mean (SD)	7.3 (2.6)	6.5 (2.8)	0.04
Adherence score (those on ULT only), mean (SD)	42.7 (2.7)	39.0 (7.4)	<0.001

BIPQ: brief illness perception questionnaire.

### Clinical and psychological factors independently associated with SU target

The factors independently associated with SU target were determined using logistic regression analysis. All factors with p < 0.15 in the univariate analysis were included in the models. In those patients prescribed ULT (n = 181), allopurinol dose, patient confidence to keep SU under control, female sex, and ethnicity were independently associated with SU target (Table 3). Adherence scores, other self-efficacy measures and illness perception scores were not independently associated with SU target.

**Table 3:** Forward stepwise logistic regression analysis of factors associated with SU target in those taking ULT

**Variable**	**Odds ratio**	**95% CI**	**p**	**Model**
Female sex	4.3	1.6-11.4	0.003	Adjusted R^2^ = 0.35, p < 0.001
Māori or Pacific ethnicity	0.19	0.07-0.52	0.001	
Allopurinol dose (per every 100 mg/day)	2.22	1.43-3.44	<0.001	
Confidence to keep serum urate under control (per every point on 0–10 Likert scale)	1.025	1.007-1.044	0.006	

Excluded from model: secondary care treatment, adherence score, age, confidence to have blood tests at recommended frequency, confidence to take gout medications regularly, BIPQ treatment control score, BIPQ understanding score.

Model included all factors with p < 0.15 between groups in univariate analysis.

### SU target and measures of gout activity

Patients at SU target reported lower gout flare frequency (p = 0.03), compared with those who were not at target (Table 4). There was a trend to less tophaceous disease in the group at SU target (p = 0.08). There were no differences between groups in HAQ-II scores or work absences.

**Table 4:** Serum urate target and measures of gout activity

	**At SU target (<0.36 mmol/L)**	**Above SU target (≥0.36 mmol/L)**	**p**
	**n = 89**	**n = 184**	
Flare frequency, mean (SD)	1.2 (2.3)	3.2 (11.4)	0.03
Presence of tophi, n (%)	9 (10%)	34 (18.5%)	0.08
HAQ-II, mean (SD)	0.46 (0.52)	0.51 (0.63)	0.46
Days off work due to gout, mean (SD)	1.2 (5.6)	0.9 (3.3)	0.52

## Discussion

Despite widespread recognition that long term SU lowering to target is required for good clinical outcomes in gout, this study has confirmed that the majority of patients with gout are not at SU target. This work has highlighted ULT prescription and allopurinol dosing as central modifiable factors associated with SU target. The data emphasize the important role of the health care professional to ensure ULT prescription at sufficient doses to reduce SU to target.

The finding that prescription and dose of allopurinol are key variables associated with SU target is consistent with previous reports from our group (in a different study population of patients with longstanding gout) and from others, showing a close relationship between allopurinol doses and SU concentrations [8,9]. Collectively, these findings provide further support for a treat-to-SU-target approach to long term allopurinol dosing [19]. A recent qualitative study of health care providers has identified barriers to effective gout management which include lack of knowledge about gout and reluctance to offer ULT as a long-term management strategy [20]. Furthermore, a ‘package of care’ intervention with close monitoring by nurse practitioners aimed to achieve serum urate target has shown excellent results in patients with gout [21]. Our data further highlight the need for education of health care professionals and changes in prescriber behaviour to optimize gout management.

Many recent studies and commentaries have focused on the need to address patient factors such as adherence and health related behaviours for optimal treatment of gout [10,22-24]. However, in our analysis of patients taking ULT, adherence was not an independent variable in the logistic regression model which included allopurinol dose. Furthermore, aside from confidence to keep SU under control, patient psychological factors and health-related behaviours were not independently associated with SU target in those taking ULT. These data do not discount the potential role of patient behaviours, but highlight the dominant role of effective ULT prescribing.

The cross-sectional design of this study does not allow conclusions to be made about the direction of the relationship between SU target and confidence to keep SU under control; that is, whether high confidence to keep SU under control reflects the patient’s experience of good control, or influences the ability to achieve target. It is possible that feedback from clinic visits in patients achieving SU target increases confidence and reinforces behaviour. Longitudinal analysis of this group is underway to further address this issue.

This study has also identified several independent non-modifiable variables associated with SU target. Women were more likely to be at target. It is possible that allopurinol doses relative to creatinine clearance may have been higher in women. However, we did not observe a relationship between creatinine clearance and SU targets in this study. Patients of Māori or Pacific ethnicity were less likely to be at target. Population based studies have demonstrated that men and those of Polynesian ancestry have higher mean SU concentrations [25,26], and high baseline levels may mean that therapeutic SU targets are more difficult to achieve. Alternatively, different health-care utilisation behaviour between different sexes or ethnicities may have contributed to the differences observed. Of interest, other clinical variables associated with higher SU concentrations such as diuretic use, body mass index and creatinine clearance were not associated with SU target.

We acknowledge the potential limitations of this study. Long-term SU lowering is recommended for optimal gout management, and this study has addressed SU target at a single timepoint. Furthermore, the cross-sectional nature of this study does not allow analysis of the direction of the relationship between SU target and other variables. However, many of the variables included in the analysis are modifiable and dynamic, and this approach allowed for direct analysis of these variables at the time that the SU was obtained. Although patients were recruited from a wide range of clinical settings, adherence and health-related behaviours may be different in those willing to participate in a research study, compared with those who are not. The study design is not able to capture an individual physician’s reasons for not escalating doses of ULT, emphasizing the importance of future studies examining why physicians do not initiate ULT or titrate appropriately. Consistent with other studies in gout, flare frequency was self-reported, and not verified by a health care professional. However, this definition is consistent with that used in other long term studies of gout [2,3].

## Conclusions

In summary, SU target is frequently not achieved in patients with gout. Greater ULT use and higher doses of allopurinol, rather than patient behaviours, are the key modifiable variables associated with suppression of SU to target. These data support interventions focusing on prescription and dosing of ULT to improve outcomes in patients with gout.

## Competing interest

The authors have no conflict of interest to declare.

## Authors’ contributions

ND (the guarantor) accepts full responsibility for the work and the conduct of the study, had access to the data, and controlled the decision to publish. ND conceived the study, participated in data analysis, and drafted the manuscript, MEH and AH recruited the patients, collected data, and assisted with the analysis, KJP assisted with study design, data analysis, and drafting the manuscript, FMM assisted with study design and drafting the manuscript, WJT assisted in study design, data analysis, and drafting the manuscript. All authors read and approved the final manuscript.

## Funding statement

This work was funded by the Arthritis New Zealand, the University of Auckland and the Henry Cotton Charitable Trust.

## Pre-publication history

The pre-publication history for this paper can be accessed here:

http://www.biomedcentral.com/1471-2474/13/174/prepub
